# Effects of Lacidophilin Tablets, Yogurt, and Bifid Triple Viable Capsules on the Gut Microbiota of Mice with Antibiotic-Associated Diarrhea

**DOI:** 10.1155/2022/6521793

**Published:** 2022-03-22

**Authors:** Ni Yang, Yang Zhan, Jianhua Wan, Yingmeng Li, Xu Hu, Wenjun Liu

**Affiliations:** Key Laboratory of Taste Correction (Taste Masking) and Sensory Evaluation of Traditional Chinese Medicine, Jiangzhong Pharmaceutical Co., Ltd., Nanchang, Jiangxi 330096, China

## Abstract

Antibiotic-associated diarrhea (AAD) is a common morbidity caused by antibiotic use and is characterized by the dysbiosis of the gut microbiota. Several clinical trials have shown that probiotics can prevent AAD. This study aimed at investigating the effects of Lacidophilin tablets (LB), yogurt (YG), and bifid triple viable capsules (BT) on the gut microbiota of mice with AAD. Mice with diarrhea were randomly allocated to treatment groups or the control group and were treated with either LB, YG, BT, or vehicle control. The body weight, diarrhea scores, cecum index, and cecal length were determined. Fecal samples of all mice were analyzed using 16S rRNA high-throughput sequencing. The results showed that LB, YG, and BT significantly decreased the diarrhea scores and inhibited increases in the cecum index and cecal length induced by AAD. In addition, they significantly changed the composition and richness of the gut microbiota. Specifically, they increased the abundance of the phylum Firmicutes and decreased the abundance of the phyla Bacteroidetes and the family Bacteroidaceae. Treatment with LB and YG also decreased the abundance of the phylum Proteobacteria and only LB could mediate the reduced levels of Lactobacillaceae in AAD mice. At the genus level, YG and BT treatment decreased the abundance of *Bacteroides* or *Parasutterella*. To our surprise, only LB treatment dramatically increased the abundance of *Lactobacillus* and decreased that of potential pathogens, such as *Bacteroides*, *Parabacteroides,* and *Parasutterella*, to almost normal values. Our findings indicate that LB, YG, and BT ameliorated diarrhea by regulating the composition and structure of the gut microbiota and that LB plays an important role in regulating the gut microbiota.

## 1. Introduction

Antibiotics are an effective treatment for many bacterial diseases resulting from pathogenic infection [1]. However, the inappropriate use of antibiotics brings about serious complications, such as antibiotic resistance, diarrhea, and increased rates of diseases related to antibiotic use, which have raised significant clinical concerns [1–4]. Antibiotic-associated diarrhea (AAD) is a common side effect of antibiotic use, affecting 5–70% of adult patients taking antimicrobial agents [5]. Broad-spectrum antibiotics (cefixime and ampicillin) can disrupt the balance of the gut microbiota, resulting in clinical symptoms of diarrhea [6, 7]. Therefore, finding alternatives to antibiotics in diarrhea treatment and antipathogenic infection has become of great concern [8].

Probiotics have been shown to reestablish the disrupted intestinal microflora and inhibit pathogens, a number of clinical trials have used probiotics for the prevention of AAD [9–12]. Many of the well-characterized probiotic strains are lactic acid bacteria (LAB), which are important starter, commensal, or pathogenic microorganisms [13]. LAB are classified in the phylum Firmicutes, order Lactobacillales, genus *Lactobacillus*, and the characteristics of LAB growth, metabolism, and production of antibacterial compounds are essential for inhibiting the growth of pathogens [14, 15]. Several meta-analyses showed a significant reduction in AAD with the use of probiotics, namely, *Lactobacillus* and *Bifidobacterium* [16–18]. A number of methods have been used to administer these probiotics, including tablets, capsules, and yogurts [16, 19, 20]. The bacteriocin preparation fermented by LAB, Lacidophilin tablets (LB), is a biologically active protein that possesses activity towards closely related Gram-positive bacteria, whereas producer cells are immune to their own bacteriocin [21]. Bifid triple viable capsules (BT) is a probiotic product mainly made of three probiotics, *Bifidobacterium longum*, *Lactobacillus acidophilus,* and *Enterococcus faecalis*, which has an excellent regulatory effect on the intestinal microenvironment [22]. However, the effects of LB, BT, and yogurt (YG) on AAD and their differences are unclear.

The gut microbiota, an organization of complex ecosystems, plays a crucial role in maintaining gut homeostasis and host health [23]. Healthy intestinal microbiota contribute to protecting the intestinal mucosa, preventing pathogens, and regulating host immunity [24]. Conversely, gut microbiota dysbiosis impairs physiological and immune functions of the human body directly or indirectly, leading to side effects such as diarrhea [25]. Studies have shown that AAD is usually accompanied by marked changes in the gut microbiota [26, 27]. However, there are no studies on the effects of LB, YG, and BT on intestinal flora disturbances caused by AAD.

Therefore, this study aimed at clarifying the role of LB, YG, and BT in AAD and its underlying mechanism. We focused on revealing the effects of LB, YG, and BT on the composition and diversity of the microbial environment in the intestine of AAD mice by 16S rRNA high-throughput sequencing.

## 2. Materials and Methods

### 2.1. Materials

Lacidophilin tablets (Cat: 19070012, each tablet contained 0.4 g Lactobacillus) were obtained from Jiangzhong Pharmaceutical Co., Ltd. (Jiangxi, China). Ambrosial yogurt containing probiotics (*Streptococcus thermophilus*, 1 × 10^4^ CFU/g) was purchased from Inner Mongolia Yili Industrial Group Co., Ltd. (Inner Mongolia, China). Bifid triple viable capsules (Cat: 04720190513) containing probiotics (*Bifidobacterium longum*, *Lactobacillus acidophilus,* and *Enterococcus faecalis*, ≥5 × 10^7^ CFU/g) were purchased from SPH Sine Pharmaceutical Laboratories Co., Ltd. (Shanghai, China). Gentamycin sulfate (potency ≥ 590 U/mg) was purchased from Fuan Pharmaceutical Group Yantai Justaware Pharmaceutical Co., Ltd. (Shandong, China). Lincomycin hydrochloride (purity ≥ 90%) was purchased from Henan Xinxiang Huaxing Pharmaceutical Factory (Henan, China).

### 2.2. Ethics Statement

Male Kunming mice weighing 20 ± 2 g (animal license number: SCXK(Xiang) 2019-0004) were purchased from Hunan SJA Laboratory Animal Co., Ltd. (Hunan, China). All mice were housed individually in a specific pathogen-free animal room at a temperature of 22 ± 0.5°C, humidity of 50 ± 5%, and a 12 h light/12 h dark cycle with free access to food and water. Animal use and procedures were conducted in accordance with the Guidelines for the Care and Use of Laboratory Animals and the Chinese Legislation on Laboratory Animals.

### 2.3. Animals and Experimental Design

After acclimatizing the animals, AAD model mice were intragastrically administered a mixture of gentamycin sulfate and lincomycin twice a day for 5 days by the following previously described procedures [27, 28]. To explore the effect of LB, YG, and BT on AAD, these mice were randomly divided into five groups of eight mice each: the normal group (NC), the AAD model group (MC), the LB treatment group, the YG treatment group, and the BT treatment group. LB, YG, and BT mice were gastric gavaged with lacidophilin tablets (1.2 g/kg/d), yogurt (0.05 g/kg/d, containing 500 CFU/kg/d), and bifid triple viable capsules (0.28 g/kg/d, containing 1.4 × 10^7^ CFU/kg/d), respectively, and NC and MC mice were treat with the same volume of physiological saline, respectively. Each mice was intragastrically treated for 10 consecutive days. Body weight was measured every fifth day during the experimental period. Diarrhea scores were used to assess the severity of diarrhea based on the previous study [29]: 0, normal stools; 1, slight diarrhea with wet and soft stools; 2, moderate diarrhea with unformed stools and mild perianal stains; and 3, severe diarrhea with watery stools and severe perianal stains. Fecal samples were collected from mice on the last day of the experiment and flash-frozen in liquid nitrogen before storage at −80°C. After fasting for 12 hours, all mice were anesthetized with an intraperitoneal injection of 1% sodium pentobarbital (0.6 ml/100 g), and the cecum was immediately collected under sterile conditions. Then, the length of the cecum [30] and the weight of cecum with contents were measured. The cecum index was calculated with the following formula [31]:

(1) $$\begin{array}{c} \text{cecum index}=\frac{\text{cecum weight}\left(g\right)}{\text{body weight}\left(g\right)}*100\%. \end{array}$$

### 2.4. DNA Extraction and Sequencing

Total microbial genomic DNA from fecal samples was extracted using the PF Mag-Bind soil DNA kit (Omega Bio-Tek, USA) according to the manufacturer's instructions. The integrity of the DNA extract was evaluated on a 1% agarose gel, and the DNA concentration and purity were determined with a NanoDrop 2000 UV-Vis spectrophotometer (Thermo Scientific, Wilmington, USA). V3–V4 region of the bacterial 16S rRNA gene was amplified with the primer pair 338F (5′-ACTCCTACGGGAGGCAGCAG-3′) and 806R (5′-GGACTACHVGGGTWTCT AAT-3′). Amplification was performed as follows: initial denaturation at 95°C for 3 min, 30 cycles of denaturation at 95°C for 30 s, annealing at 55°C for 30 s, extension at 72°C for 45 s, a single extension at 72°C for 10 min, and holding at 4°C. PCR products were confirmed using 2% agarose gel electrophoresis and subsequently purified with an AxyPrep DNA gel extraction kit (Axygen Biosciences, Union City, CA, USA). After purification, the PCR products were quantified with a Quantus™ fluorometer (Promega, USA) and were then sequenced with an Illumina MiSeq sequencing platform (Illumina, San Diego, CA, USA). Sequencing was completed by Shanghai Majorbio Bio-Pharm Technology Co., Ltd. (Shanghai, China).

### 2.5. Bioinformatics Analysis

QIIME (version 1.9.1) was used for quantitative analysis of raw DNA sequences. Operational taxonomic units (OTUs) with a 97% similarity cutoff were clustered using UPARSE (version 7.1, http://drive5.com/uparse/), and chimeric sequences were removed. The taxonomy of each OTU representative sequence was classified using the ribosomal database project (RDP) classifier (http://rdp.cme.msu.edu/) with a 0.7 confidence value as the cutoff. Alpha diversity indices, including Shannon, Simpson, ACE, and Chao1, were calculated by MOTHUR (version v.1.30.2, https://www.mothur.org/wiki/Download_mothur), and the corresponding rarefaction curves were generated with R software (version 3.5). Beta-diversity analyses were conducted to investigate structural variations in the microbial communities across the samples using principal component analysis (PCA) [32]. Statistical analysis of the sample microbial community structure at the phylum, family, and genus levels was carried out via QIIME. Furthermore, the OTU abundance was normalized, including the removal of 16S rRNA marker gene copy numbers and calculation of cluster of orthologous groups of proteins (COG) via PICRUSt software [33].

### 2.6. Statistical Analysis

The SPSS 19.0 (IBM-SPSS Inc., Chicago, Illinois, USA) statistical software package was used for all data analyses, and the results are expressed as the mean ± standard deviation (SD). Statistical significance was determined by using one-way analysis of variance (ANOVA) for three group comparisons and the Wilcoxon rank sum test for multiple group comparisons. Differences were considered significant with a *p* value < 0.05 or a *p*-value < 0.01.

## 3. Results

### 3.1. Effect of LB, YG, and BT on Body Weight, Diarrhea Scores, Cecum Index, and Cecal Length in Mice

**Figure 1:**
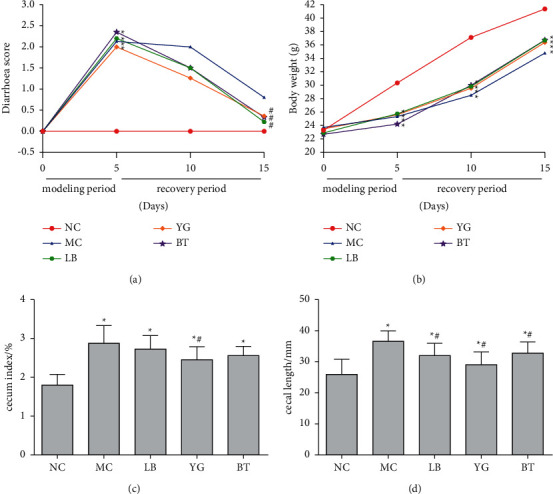
Effect of LB, YG, and BT on diarrhea score (a), body weight (b), cecum index, (c) and cecal length (d) of mice (*n* = 8). NC, normal group; MC, model group; LB, Lacidophilin tablet group; YG, yogurt group; BT, bifid triple viable capsule group. Values are represented as the mean ± SD. ^*∗*^*p* < 0.05 compared with the normal group, and ^#^*p* < 0.05 compared with the model group.

As shown in Figure 1, during the establishment period of the model, compared with the NC group, diarrhea scores of the mice increased significantly (*p* < 0.05), the body weight of mice decreased significantly (*p* < 0.05), and watery stools and perianal stains indicate the successful establishment of the AAD mice model, which mirrors the findings of Xu et al. [34] in the 5-fluorouracil-induced diarrhea mice. After treatment with LB, YG, and BT, the diarrhea scores were gradually decreased, and the rate of increasing body weight was higher than that during the diarrhea period (Figures 1(a) and 1(b)). Meanwhile, the results also showed that MC mice had a significantly increased cecum index (2.87 ± 0.46) compared with NC mice (1.80 ± 0.28) (*p* < 0.05, Figure 1(c)). LB, YG, and BT administration reduced the antibiotic enlargement of the cecum, and the cecum index of the mice in the LB, YG, and BT groups decreased by 6, 17, and 12% respectively, when compared to that of the MC group. Furthermore, the MC group exhibited a significant increase in the cecal length by 41% compared to that of the NC group (*p* < 0.05, Figure 1(d)), while the LB, YG, and BT groups showed a significant decrease by 15%, 21%, and 12%, respectively. And there was no significant difference between mice in the LB, YG, and BT groups in terms of body weight, cecum index, or cecal length (*p* > 0.05). These data indicated that LB, YG, and BT were efficient in improving AAD.

### 3.2. Analysis of Microbial Community Structure and Diversity

**Figure 2:**
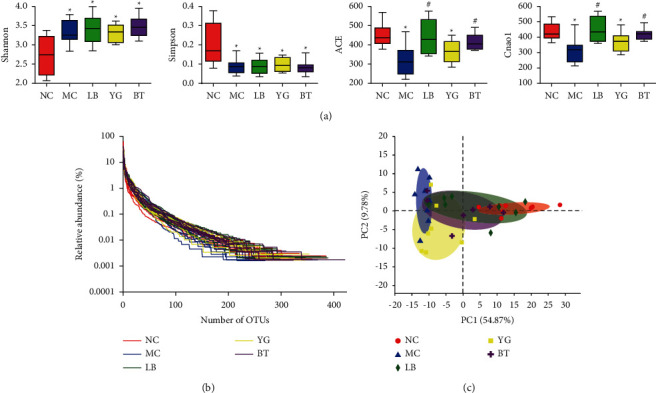
Changes in the gut microbiota diversity of different groups. Alpha diversity indices including Shannon, Simpson, ACE, and Chao1 indices (a); (b) rank-abundance curve; (c) PCA score plot of the microbiota in the different groups. NC, normal group; MC, model group; LB, Lacidophilin tablet group; YG, yogurt group; BT, bifid triple viable capsule group. Values are represented as the mean ± SD. ^*∗*^*p* < 0.05 compared with the normal group, and ^#^*p* < 0.05 compared with the model group.

In this study, 1,887,722 high-quality sequences and 1,211 OTUs were obtained from samples. The coverage of each sample reached 99.9%, indicating that the sequences could reflect the actual situation of intestinal bacteria in the samples [28]. Alpha diversity indices reveal the diversity and richness of microbial communities. The results revealed remarkable increases in community diversity (Shannon and Simpson) and decreases in community richness (ACE and Chao1) indices in the MC group compared to the NC group (*p* < 0.05). LB and BT significantly increased the ACE and Chao1 indices (*p* < 0.05 vs. MC group). However, the Shannon and Simpson indices in the LB, YG, and BT groups did not show a visible difference compared to the MC group (Figure 2(a)). Moreover, rarefaction analyses indicated that the sequencing depth of the gut microbial environment was adequately captured in samples and suitable for further analysis (Figure 2(b)). For beta-diversity analysis, PCA was used to measure the similarity of sample compositions [3]. A PCA score plot showed that the gut microbiota was markedly altered in the MC group, which had a distinct cluster far away from that of the NC group, and the LB and BT groups were closer to the NC group than to the MC group. The variations explained by PC1 and PC2 were 54.87% and 9.78%, respectively (Figure 2(c)).

### 3.3. Composition Analysis of the Gut Microbiota

**Figure 3:**
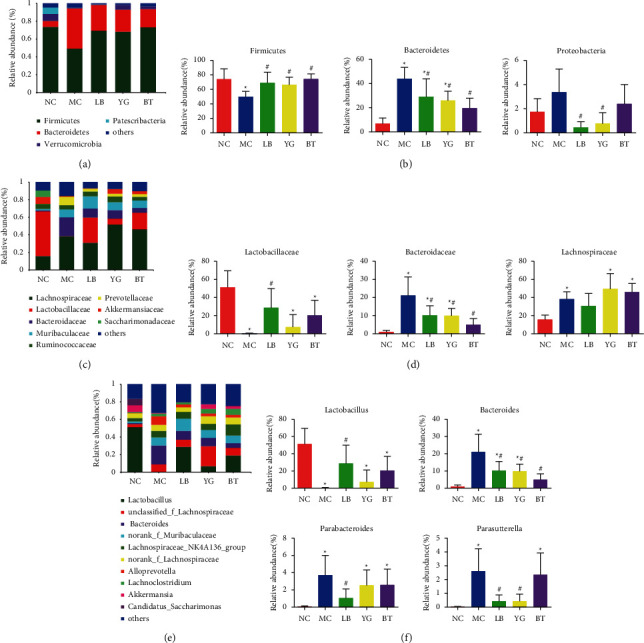
The composition of gut microbiota in different groups. (a) Phylum level; (b) main differences in compositions at the phylum level; (c) family level; (d) main differences in compositions at the family level; (e) genera level; (f) main differences in compositions at the genus level. NC, normal group; MC, model group; LB, Lacidophilin tablet group; YG, yogurt group; BT, bifid triple viable capsule group. Values are expressed as the mean ± SD. ^*∗*^*p* < 0.05 compared with the normal group; ^#^*p* < 0.05 compared with the model group.

The community bar indicates the abundance levels of various phyla, family, and genera in different groups (Figure 3). At the phylum level, all the groups were mainly composed of Firmicutes, Bacteroidetes, and Proteobacteria (Figure 3(a)). Antibiotic treatment induced a significant decrease in the relative abundance of Firmicutes and a dramatic increase in that of Bacteroidetes compared to the control group (*p* < 0.05, Figure 3(b)). After LB, YG, and BT treatments, the relative abundances of Firmicutes and Bacteroidetes were restored to their respective normal levels (*p* < 0.05 vs. MC group). In addition, the relative abundance of Proteobacteria had a slight variation in different groups, with a decreased abundance in the LB and YG groups (*p* < 0.05, Figure 3(b)). There was no significant difference in the main components between the LB, YG, and BT groups at the phylum level. At the family level, antibiotic treatment induced a significant decrease in the relative abundance of Lactobacillaceae and a dramatic increase in that of Bacteroidaceae and Lachnospiraceae compared to those of the control group (*p* < 0.05, Figures 3(c) and 3(d)). And the treatment of LB, YG, and BT decreased the relative abundance of Bacteroidaceae (*p* < 0.05), whereas only LB, but not YG or BT, increased the relative abundance of Lactobacillaceae significantly (*p* < 0.05, Figures 3(c) and 3(d)).

At the genus level, the relative abundances of *Lactobacillus*, *Bacteroides*, *Parabacteroides,* and *Parasutterella*, with a higher ratio were 51.35%, 1.03%, 0.064%, and 0.017% in the NC group, respectively (Figure 3(e)). *Lactobacillus* was the predominant genus in all samples. In mice from the MC group, there was a decrease in the abundance of *Lactobacillus* but an increase in the abundance of *Bacteroides*, *Parabacteroides,* and *Parasutterella* compared with mice from the NC group (*p* < 0.05, Figure 3(f)). As shown in Figure 3(f), from the MC group to the LB group, the relative *Lactobacillus* abundance increased from 0.24% to 29%, and the relative *Parabacteroides* abundance decreased from 3.72% to 1.03% (*p* < 0.05), with no significant difference compared to that in the NC group (*p* > 0.05). Neither YG nor BT could inhibit the changes in these genera caused by AAD. Additionally, compared with the MC group, the LB, YG, and BT groups exhibited significantly decreased relative abundances of *Bacteroides* (*p* < 0.05), with no significant difference between those of the BT and NC groups. Moreover, both the LB and YG groups exhibited significantly decreased relative abundances of *Parasutterella* (*p* < 0.05), and a noticeable change was not observed in the BT group.

### 3.4. Functional Analysis of the Gut Microbiota

**Figure 4:**
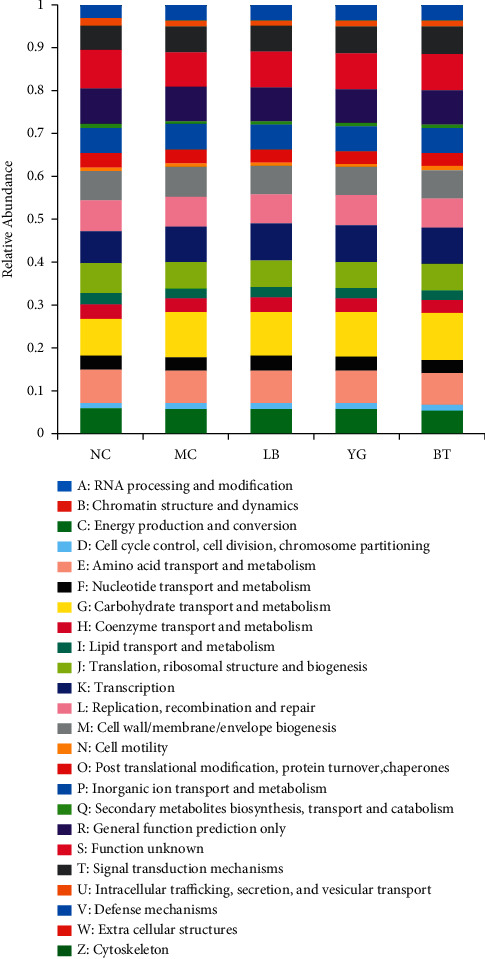
COG function classification analysis of different groups. The relative abundance was calculated using the average abundances via PICRUSt. NC, normal group; MC, model group; LB, Lacidophilin tablet group; YG, yogurt group; BT, bifid triple viable capsule group.

To further investigate changes in the functional profiles of intestinal bacteria in response to gut community changes, we analyzed the gene function pattern using PICRUSt software. As shown in Figure 4, the results of COG functional enrichment analysis indicated that the intestinal flora functions related to AAD were mainly enriched in carbohydrate transport and metabolism, amino acid transport and metabolism, transcription and replication, recombination, and repair.

## 4. Discussion

It is well known that most causes of diarrhea are related to the imbalance of intestinal microbiota and colonization of pathogenic microorganisms in the gastrointestinal tract [35]. Several probiotics may prevent AAD by inhibiting pathogens, restoring the gut microflora, and other potential mechanisms of action [36–38]. In this study, we used AAD mice to identify the effect of Lacidophilin tablets, yogurt, and bifid triple viable capsules in diarrhea. The results showed that LB, YG, and BT significantly inhibited the increase in the diarrhea scores, cecum index, and cecal length, as previously reported [39–41], indicating positive effects on alleviating diarrhea.

Then, the response of gut microbiota to diarrhea and LB, YG, and BT treatment was determined with 16S rRNA analysis. Previous reports have shown that probiotics reshape the gut microecology of individuals recovering from antibiotic treatment by changing the composition of intestinal bacteria [4] and the gut microbiota failed to recover its initial state despite the alleviation of diarrhea [42]. Interestingly, although LB, YG, and BT could alleviate diarrhea, the remarkable variation in gut microbiota was different in these groups. As demonstrated previously [43, 44], gut microbiota analysis revealed that AAD mice possess lower alpha diversity than control mice, and it is probably because most of the mice were suffering from diarrhea during the experiment. Attractively, after the treatment of LB and BT, the alpha diversity of diarrheal mice was restored to the levels of control ones, while the Shannon and Simpson indices were not significantly changed, which may be caused by the stability of the majority of the gut microbiome [34]. And Shade et al. pointed out that diversity is not good or bad; it is a starting point for further inquiry of ecological mechanisms rather than an “answer” to community outcomes [45]. Consistent with the results of alpha diversity analysis, a significant difference was detected in the principal components of the microbial community structure in diarrheal and control mice by PCA, and LB and BT treatment homogenized the gut microbiota so that the gut microbiota of mice was similar to that of control ones. Therefore, we speculated that LB and BT can repair the gut microbiota of diarrheal mice and make their composition similar to that of control mice.

Furthermore, we figured out the key phylotypes of gut microbiota modulated by LB, YG, and BT treatment. At the phylum level, Firmicutes, Bacteroidetes, and Proteobacteria were the three dominant bacterial phyla in the feces of control mice in our study. Previous studies have reported that Firmicutes was often highly represented in the gut microbiota of healthy individuals and could be reduced with illness, while a significant increase in Proteobacteria abundance could lead to chronic abdominal pain/diarrhea and a series of gastrointestinal inflammations [46–48]. The phylum Bacteroidetes comprises bacteria that are common gut-associated microbes with a relatively high risk of causing diarrheal diseases and had a negative correlation with inflammatory cytokines [49, 50]. The ratio of Firmicutes/Bacteroidetes is commonly used to evaluate various enteropathies of patients, such as irritable bowel syndrome, inflammatory bowel disease, and metabolic diseases [51, 52]. Hu et al. reported that protocatechuic acid, ferulic acid, and vanillic acid could increase the Firmicutes/Bacteroidetes ratio in the weaned piglet model, which was associated with inflammation and intestinal barrier function [53, 54]. Our present study showed that AAD not only decreased Firmicutes abundance but also increased Bacteroidetes and Proteobacteria abundance. In addition, LB and YG treatment restored the abundance of Firmicutes, Bacteroidetes, and Proteobacteria to nearly normal levels, while BT treatment could only reduce the abundance of Firmicutes and Bacteroidetes, which may be caused by their different compositions. Moreover, the family Lachnospiraceae, one of the producers of short-chain fatty acids, was significantly increased in IBS-D. It has been demonstrated that the Lachnospiraceae taxa could contribute to the general symptoms of IBS [55, 56]. Jia et al. [57] reported that the abundance of Bacteroidaceae increased significantly and Lactobacillaceae decreased significantly in the DSS-treated mice, and our results were consistent with this finding. Recent studies have also found that intestinal dysbacteriosis was the leading cause of infantile diarrhea and Lactobacillaceae could alleviate the severity of diarrhea [58, 59]. In our study, the treatment of LB, YG, and BT could significantly decrease the abundance of Bacteroidaceae, while only LB had a significant positive effect on the Lactobacillaceae level.

At the genus level, AAD mice exhibited high abundances of *Bacteroides*, *Parabacteroides,* and *Parasutterella* but a low abundance of *Lactobacillus* compared to control mice, which was in agreement with literature reports [3, 27]. Among these, *Lactobacillus* showed the highest overall relative abundance in all groups, which is consistent with a previous study [27]. Susanne et al. reported that *Lactobacillus* interventions were associated with a reduction in AAD [60]. Lact*obacillus* strains could modulate the gastrointestinal microbiota, reduce the number of harmful bacteria, and enhance the host immune system [61]. In the current study, to our surprise, only LB treatment reversed the decreased abundance of *Lactobacillus* caused by AAD to near-normal levels. *Bacteroides* is a pivotal pathogenic bacterium associated with AAD and belongs to the Bacteroidetes phylum [62]. It has been reported that *Bacteroides* play an important role in breaking down complex molecules within the intestine and function to assist the body's immune system in fighting against potentially harmful pathogens [63]. Our study showed that treatment with LB, YG, and BT significantly reduced the increased abundance of *Bacteroides* caused by AAD. *Parabacteroides* is considered an opportunistic pathogen that is frequently involved in infectious diseases, mainly intra-abdominal processes and bacteremia, and develops resistance to antimicrobial drugs [64]. High-abundance *Parabacteroides* members are more likely to cause chronic inflammatory disorders and even colorectal cancer [65, 66]. Our findings indicated that only LB treatment could decrease the *Parabacteroides* abundance to near that of normal mice. Furthermore, the genus *Parasutterella* has been defined as a member of the healthy fecal core microbiome in the human gastrointestinal tract [67]. Previous reports have shown that *Parasutterella* is associated with dysbiosis, a decrease in intestinal flora diversity, and even the development and progression of IBS [68, 69]. In this study, we found that both LB and YG treatments could obviously prevent the increased abundance of *Parasutterella*. These findings suggest that LB, rather than YG or BT, was more capable of reversing AAD-induced changes in key phylotypes of gut microbiota. The beneficial effects of LB, YG, and BT on AAD were correlated with their regulatory activity on gut microbiota composition and diversity. However, their regulation of intestinal flora showed a large difference, of which LB intervention indicated the primary effect, consistent with a previous study [60]. The difference may be caused by their various gut bacterial compositions and processing technologies, the LB is a metabolite production of LAB, and BT and YG are different probiotics with the live bacteria, which affect gut microbial metabolism and pharmacokinetics in vivo [70]. Briefly, the underlying mechanisms of the different effects of LB, YG, and BT on gut microbiota are still worth studying. We hope to address these mechanisms in our future research.

## 5. Conclusions

In summary, we applied 16S rRNA gene high-throughput sequencing to elucidate the mechanism of LB, YG, and BT improvement of AAD in mice. Our study indicated that all LB, YG, and BT treatments could effectively alleviate AAD and positively affected the microbial environment in the gut of mice with AAD. Interestingly, we demonstrated that compared with YG and BT administration, LB treatment had the strongest regulatory effect on gut microflora, including significantly enhanced microbial diversity and marked changes in the gut microbiota structure, and the reason for these differences in LB, YG, and BT treatment requires further investigation. Collectively, LB, YG, and BT altered the gut microbiota composition in mice with AAD, increased its richness, and promoted the reestablishment of the gut microbial environment, thus alleviating the symptoms of diarrhea.

## Data Availability

All the data that were used to support the findings of this study are included within the article.
